# The Role of Minimally Invasive Techniques in Scoliosis Correction Surgery

**DOI:** 10.1155/2018/4185840

**Published:** 2018-01-24

**Authors:** Michael B. Cloney, Jack A. Goergen, Angela M. Bohnen, Zachary A. Smith, Tyler Koski, Nader Dahdaleh

**Affiliations:** ^1^Department of Neurological Surgery, Feinberg School of Medicine, Chicago, IL, USA; ^2^Feinberg School of Medicine, Chicago, IL, USA

## Abstract

**Objective:**

Recently, minimally invasive surgery (MIS) has been included among the treatment modalities for scoliosis. However, literature comparing MIS to open surgery for scoliosis correction is limited. The objective of this study was to compare outcomes for scoliosis correction patients undergoing MIS versus open approach.

**Methods:**

We retrospectively collected data on demographics, procedure characteristics, and outcomes for 207 consecutive scoliosis correction surgeries at our institution between 2009 and 2015.

**Results:**

MIS patients had lower number of levels fused (*p* < 0.0001), shorter surgeries (*p* = 0.0023), and shorter overall lengths of stay (*p* < 0.0001), were less likely to be admitted to the ICU (*p* < 0.0001), and had shorter ICU stays (*p* = 0.0015). On multivariable regression, number of levels fused predicted selection for MIS procedure (*p* = 0.004), and multiple other variables showed trends toward significance. Age predicted ICU admission and VTE. BMI predicted any VTE, and DVT specifically. Comorbid disease burden predicted readmission, need for transfusion, and ICU admission. Number of levels fused predicted prolonged surgery, need for transfusion, and ICU admission.

**Conclusions:**

Patients undergoing MIS correction had shorter surgeries, shorter lengths of stay, and shorter and fewer ICU stays, but there was a significant selection effect. Accounting for other variables, MIS did not independently predict any of the outcomes.

## 1. Introduction

Adult scoliosis is a spinal deformity typically caused by asymmetrical disc degeneration, osteoporosis, and vertebral body compression fractures [1]. When nonsurgical treatment fails, there are multiple surgical techniques that can be used [2]. The goals of surgery are to improve functionality, relieve pain, improve cosmesis, and prevent curve progression [3]. Whether performed posteriorly or anteriorly, open techniques are associated with large blood loss, muscle injury and denervation, significant postoperative pain, and other complications [4, 5].

Minimally invasive surgery (MIS) potentially avoids or lessens these complications due to its ability to reduce intraoperative blood loss, soft tissue damage, infection, postoperative pain, and recovery time [6]. The safety and feasibility of MIS for adult degenerative scoliosis have already been established [7]. Also, results have previously been reported that showed similar clinical improvement for patients who underwent open surgery versus MIS [8]. Furthermore, the patients who underwent MIS had lower morbidity and complication rates and significantly shorter hospital stays [8].

While these initial results are promising, these studies were on small subsets of patients with many confounding variables. The literature comparing open surgery versus MIS for scoliosis correction is limited; therefore, the need exists for further investigation to determine the efficacy of MIS. Here, we compared MIS and open scoliosis surgery with respect to selection for surgical technique and outcomes, including readmission rates, reoperation rates, bleeding, and clotting complications.

## 2. Methods

### 2.1. Patient Population

Patients were identified using the Northwestern University Electronic Data Warehouse (EDW). The EDW is an institution-specific registry clinical data repository jointly funded by Northwestern Memorial Hospital (NMH), Northwestern Medical Faculty Foundation (NMFF), and Northwestern University Feinberg School of Medicine. We identified all patients who underwent surgery for scoliosis in the Departments of Neurological Surgery or Orthopedic Surgery at Northwestern University between January 1, 2009, and May 31, 2015, as determined by the preoperative indication for surgery provided by the surgeon.

### 2.2. Clinical and Demographic Data

Data on patients' age, sex, race, BMI, smoking status (ever smoker versus never smoker), number of comorbid diagnoses, and insurance type (private versus other, including Medicare, Medicaid, and disability insurance) at the time of presentation were retrospectively collected for analysis. Data were also collected on the number of levels fused and length of surgery, as well as whether the patients' scoliosis correction involved a staged procedure, interbody fusion, laminectomy, or osteotomy. Data on surgical techniques and approaches used were also collected. The approaches used included lateral, posterior, and anterior. Of the 14 minimally invasive operations, most utilized an interbody fusion with percutaneous screws; see Table 1 for details.

### 2.3. Outcome Measures

Information about complications within 30 days after the surgery included the cumulative 30-day incidence and timing of VTEs (defined as either DVT or PE), all-cause readmissions, reoperations, ICU admission, length of ICU stay, length of hospital stay, and incidence of death.

### 2.4. Statistical Methods

Microsoft Excel 2011 (Microsoft, Redmond, WA, USA) was used to manage data. Statistical analysis was performed using Stata 12.0 (StataCorp, College Station, TX, USA) and Prism 6.0b (GraphPad Software, Inc., La Jolla, CA, USA). Parametric data was given as mean ± standard deviation and compared using a* t-*test. Nonparametric data was compared using Mann–Whitney *U* test or Chi-square test, as appropriate. Regression analysis was performed using stepwise logistic regression, with an inclusion threshold for the multivariable model of *p* < 0.10 for candidate variables on single-variable logistic regression. A value of *p* < 0.05 was considered statistically significant.

## 3. Results

### 3.1. Demographic Characteristics

There was no difference in age (64.5 ± 1.4 versus 58.1 ± 1.2, Δ−6.44 [−15.11, 2.22], *p* = 0.1442), gender (35.7% male versus 25.9% male, 1.588889 [0.5081890, 4.967774], *p* = 0.5304), race (*p* = 0.3243), insurance type (*p* = 0.6694), smoking status (*p* = 0.4284), BMI (26.1 ± 0.6 versus 26.8 ± 0.4, Δ0.72 ± 1.47 [−2.17, 3.61], *p* = 0.6242), or comorbid disease burden (*p* = 0.4499). On multivariable regression, age (OR 1.076323 [0.9977851, 1.161042], *p* = 0.057) and having private insurance (OR 3.735077 [0.9058839, 15.40021], *p* = 0.068) showed trends toward selection for MIS surgery (see Table 2).

### 3.2. Procedure Data

**Figure 1:**
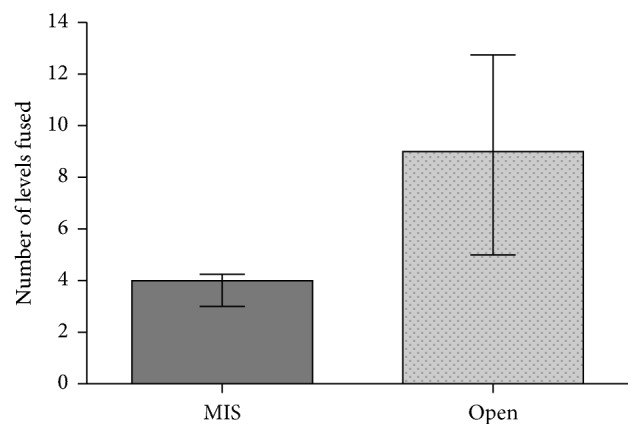
Number of levels fused for patients undergoing MIS approach versus open approach.

MIS patients were equally likely to have staged surgery (OR 0.6507177 [0.1749019, 2.420978], *p* = 0.5186), decompression (OR 0.3597285 [0.04548798, 2.844809], *p* = 0.3127), osteotomy (OR 0.2604895 [0.03313165, 2.048035], *p* = 0.1703), and allograft (OR 0.6491885 [0.2098068, 2.008732], *p* = 0.4505). There was a trend toward significance in surgical approach (*p* = 0.0857) and surgery involving the thoracic spine (OR 0.1539582 [0.008965574, 2.643795], *p* = 0.0805). MIS patients were less likely to have autograft (OR 0.1382386 [0.01769585, 1.079909], *p* = 0.0289) and had a lower number of levels fused (4.0 versus 9.0, Δ5.0 [2.0, 7.0], *p* < 0.0001, Figure 1). There was significantly more variance in the number of levels fused among patients undergoing open surgery (*p* < 0.0001). On multivariable regression, the number of levels fused predicted selection for MIS procedure (OR 0.6079009 [0.4340611, 0.8513629], *p* = 0.004), and there was a trend toward significance for selection for MIS among patients undergoing a posterior approach (OR 3.43426 [0.8365153, 14.09913], *p* = 0.087) and not requiring surgical decompression (OR 0.1319887 [0.0147237, 1.183196], *p* = 0.070) (see Table 3).

### 3.3. Outcomes for MIS versus Open Scoliosis Correction

**Figure 2:**
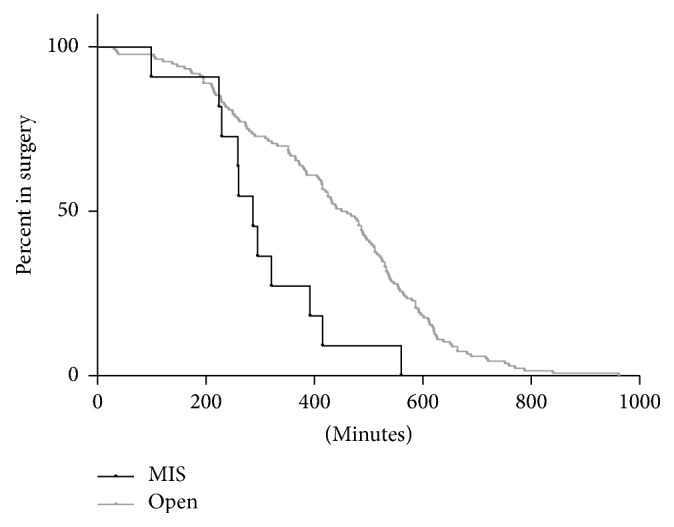
Comparison in surgery length between MIS and open approach patients.

**Figure 3:**
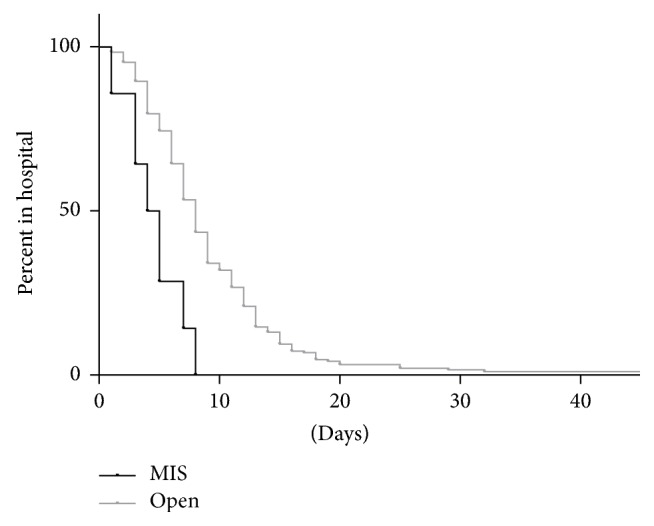
Comparison in LOS between MIS and open approach patients.

MIS surgery was significantly shorter (287.0 minutes versus 433.0 minutes, HR 2.319 [1.604, 8.342], *p* = 0.0023, Figure 2) and was less likely to last ≥6 hours (OR 0.2280405 [0.0751441, 0.6920369], *p* = 0.0051). MIS patients had shorter overall lengths of stay (4.5 days versus 8.0 days, HR 3.032 [3.725, 22.61], *p* < 0.0001, Figure 3), were less likely to be admitted to the ICU (OR 0.08779576 [0.02348702, 0.3281854], *p* < 0.0001), and had shorter ICU stays (19.0 hours versus 48.5 hours, HR 5.174 [5.200, 866.7], *p* = 0.0015).

On single-variable analysis, MIS patients were equally likely to experience the following within 30 days of surgery: readmission (OR 0.3271202 [0.01872778, 5.713847], *p* = 0.2318), reoperation (OR 1.181818 [0.06218783, 22.45929], *p* = 1.0000), DVT (OR 0.3477832 [0.01986991, 6.087253], *p* = 0.2464), PE (OR 3.634615 [0.3782204, 34.92786], *p* = 0.2328), any VTE (OR 0.8509616 [0.1044311, 6.934095], *p* = 0.8800), and postoperative death (OR 2.668966 [0.1222150, 58.28564], *p* = 0.7034). MIS patients were less likely to require transfusion (OR 0.1231231 [0.02681325, 0.5653661], *p* = 0.0017).

### 3.4. Predictors of Outcomes after Scoliosis Correction

On multivariable regression, BMI predicted DVT within 30 days postoperatively (OR 1.130749 [1.021063, 1.252219], *p* = 0.018), and age (OR 1.048752 [0.9975131, 1.102623], *p* = 0.063) showed a trend toward significance. Number of levels fused showed a trend toward significance in predicting PE 30 days postop (OR 1.251054 [0.9814253, 1.594758], *p* = 0.071). Age (OR 1.053943 [1.000293, 1.11047], *p* = 0.049) and BMI (OR 1.143371 [1.037006, 1.260645], *p* = 0.007) predicted VTE within 30 days postop. Comorbid disease burden predicted readmission within 30 days postop (OR 2.543268 [1.376737, 4.698219], *p* = 0.003), and involvement of the thoracic spine showed a trend toward significance (OR 0.1084136 [0.0109545, 1.072934], *p* = 0.057). Number of levels fused predicted prolonged surgery (surgery > 6 h) (OR 1.452142 [1.233844, 1.709062], *p* < 0.001), and a history of smoking showed a trend toward significance (OR 0.0040854 [7.33*e* − 06, 2.275654], *p* = 0.088). Number of levels fused (OR 1.297174 [1.182993, 1.422377], *p* < 0.001) and comorbid disease burden (OR 1.297174 [1.182993, 1.422377], *p* < 0.001) predicted the need for transfusion, and osteotomy showed a trend toward significance (OR 2.359625 [0.9492208, 5.865686], *p* = 0.065). Age (OR 0.9512098 [0.9089738, 0.9954083], *p* = 0.031), gender (OR 3.299076 [1.06138, 10.25448], *p* = 0.039), comorbid disease burden (OR 1.686387 [1.123766, 2.530687], *p* = 0.012), number of levels fused (OR 2.089387 [1.615015, 2.703095], *p* < 0.001), and undergoing a staged procedure (OR 5.321398 [1.470397, 19.25826], *p* = 0.011) all predicted ICU admission.

## 4. Discussion

Minimally invasive surgical techniques could potentially reduce the morbidity associated with traditional open surgical techniques in scoliosis correction [9–11]. Currently, the literature on MIS for scoliosis correction is limited. Many of the studies performed to date observed relatively few patients, and multiple systematic reviews have concluded that more research is needed [9, 12]. Our study examined 207 scoliosis correction surgeries and identified selection factors for MIS, how MIS outcomes compare to open surgery outcomes, and predictors of outcomes for each technique.

Importantly, the median number of levels fused predicted selection for MIS technique with MIS patients having fewer levels fused (4 versus 9, *p* < 0.0001). Many previous studies did not search for a selection effect for MIS versus open surgery selection. One meta-analysis on scoliosis correction, by Dangelmajer et al., did examine selection bias and found that both older patients and patients with less severe deformities were more likely to be selected for an MIS technique [13]. Our analysis agrees with this finding.

Furthermore, having private insurance (*p* = 0.068), undergoing a posterior approach (*p* = 0.087), and not requiring surgical decompression (*p* = 0.070) each showed a trend toward selection for MIS. Similarly to our finding that private insurance was an important determinant of surgery choice, a study by Park and Ha revealed that the cost of the MIS technique determined which patients underwent MIS versus open technique [6]. Another group separated the patients by allocating the private hospital patients to receive MIS and the public hospital patients in their study to receive the open technique [14]. This trend of selection biases between the two groups was consistent across most of the studies that reported patient selection information, limiting the ability to conclude a true difference in outcomes between the MIS and open techniques.

In our study, there was significantly more variance in the number of levels fused among patients undergoing open surgery (*p* < 0.0001). Dangelmajer et al. came to the same conclusion in their systematic review and attributed it to the fact that patients undergoing open procedure had a larger preoperative scoliosis [13]. This result shows that an open technique can be used for a broader range of spinal levels than MIS.

MIS surgery was significantly shorter (287.0 minutes versus 433.0 minutes, *p* = 0.0023, Figure 2) and patients undergoing MIS were less likely to have surgery last >6 hours (*p* = 0.0051) based on single-variable analysis. Anand et al. noted that their surgical outcomes data for MIS scoliosis correction was similar to open correction outcomes data when compared to the literature [8]. However, a meta-analysis on MIS versus open approach in degenerative lumbar disease revealed significant variability in operating times [15]. For example, one study found an average operating time of 161 minutes for the MIS approach compared to 375 minutes for the open approach [16]. In contrast, a study in the same meta-analysis found an average operating time of 159.2 minutes for the MIS approach versus 113.06 minutes for the open approach [17]. We suspect that confounding variables have an important impact on operating time, which would explain the significant variability between studies.

MIS patients also had shorter overall lengths of stay based on single-variable analysis (4.5 days versus 8.0 days, *p* < 0.0001, Figure 3). While it seems promising that MIS patients typically had a shorter length of stay, the results from other studies are variable, potentially indicating a selection effect [6, 14, 18]. Our length of stay results were consistent with a meta-analysis by Phan et al. that found a median length of stay of 4.7 days for the MIS approach and 8 days for the open approach [15]. Although our results are consistent with the meta-analysis, the presence of confounding variables makes it uncertain if MIS versus open surgery is the etiology of the variability in length of stay.

Compared to patients undergoing open surgery, MIS patients were less likely to be admitted to the ICU (*p* < 0.0001) but did have shorter ICU stays when it was required (19.0 hours versus 48.5 hours, *p* = 0.0015). These results are consistent with previous groups who found that open surgery patients typically had more complications following surgical treatment [13, 19]. However, similarly to our study, these groups also noted the presence of confounding variables such as age and preoperation severity of deformity that could have attributed to these results. In fact, in our analysis, open surgery did not predict ICU admission on multivariable regression.

While MIS or open correction was not independently associated with ICU admission, the number of levels fused did independently predict ICU admission (*p* < 0.001). As the number of levels fused was also independently associated with the selection of technique, it may be a confounding factor that accounts for the significant difference in likelihood of ICU admission on single-variable analysis. Similarly, age was likely a confounding factor, as age showed a trend toward significance in predicting technique, and was a significant predictor of ICU admission (*p* = 0.031).

Our finding that comorbid disease burden independently predicted ICU admission (*p* = 0.012) and readmission within 30 days (*p* = 0.003) is consistent with the existing spine surgery literature [20, 21]. Cardiac, GI, and respiratory issues that were present before the operation are frequent causes of ICU admission and readmission and appear to be an important factor when comparing MIS versus open technique for scoliosis correction as well.

On multivariable regression analysis, age (*p* = 0.049) and BMI (*p* = 0.007) predicted VTE within 30 days postop. These variables are typically found to be strongly associated with such outcomes in spinal surgery, as evidenced in numerous previous studies [22–24]. Importantly, MIS surgery was not found to be an independent predictor of any outcome analyzed during multivariable regression. So, although we found that the typical outcome predictors (age, BMI, and comorbid disease burden) were significant in this study, we did not find any significant difference in patient outcome based on MIS versus open technique alone.

Our study has a number of important limitations. The study was conducted retrospectively and is subject to the biases inherent to this study design. A prospective study would enable us to further understand if the trends we discovered (private insurance, number of levels fused showing selection for MIS) were actively affecting the surgeon's decision whether to use an MIS or open approach. The operations we collected data on varied in the minimally invasive technique and approach used, which made it a less homogenous population to draw conclusions from. As a single-institution study, it only reflects the clinical decision-making of our spine surgeons with respect to patient selection and management. Our series is limited by its size, and a larger series would allow for a more thorough comparison between MIS and open surgery. Our study does not provide radiographic comparisons of corrections, as is common in the scoliosis literature. However, multiple prior studies have compared radiographic outcome for MIS and open scoliosis correction, the results of which have been meta-analyzed [2, 5, 7, 13, 25–27]. Despite its limitations, our study contributes to the existing literature on scoliosis correction by examining selection factors for MIS versus open surgery, as well as a variety of perioperative outcomes.

## 5. Conclusion

Patients undergoing MIS scoliosis correction had shorter surgeries, shorter lengths of stay, and shorter and fewer ICU stays, but there was a significant selection effect. Accounting for other clinical variables, undergoing MIS surgery did not independently predict any of the outcomes analyzed.

## Figures and Tables

**Table 1:** Approaches and techniques used in minimally invasive operations.

	Number of operations using technique
Anterior approach	2
Posterior approach	11
Lateral approach	1
Percutaneous screws	9
Interbody fusion	10

**Table 2:** Patient demographic data for MIS approach versus open approach.

	MIS patients	Open patients	*p* value
Age	64.5 years (mean)	58.1 years (mean)	0.1442
Gender	35.7% male	25.9% male	0.5304
Race	100% Caucasian	86.01% Caucasian, 4.15% African American, 9.84% other	0.3243
Insurance type	10 private, 4 Medicare	108 private, 77 Medicare, 7 Medicaid, 1 other	0.6694
Smoking status	11 never smoked, 3 quit more than 12 months ago	119 never smoked, 53 quit more than 12 months ago, 21 current smokers or smoked within last 12 months	0.4284
BMI	26.1 (mean)	26.8 (mean)	0.6242
Comorbidities	2.43 comorbidities per patient (cardiac, renal, pulmonary, endocrine, or hypertension)	2.8 comorbidities per patient (cardiac, renal, pulmonary, endocrine, or hypertension)	0.4499

**Table 3:** Surgical procedure data for MIS approach versus open approach.

	Odds ratio for MIS patients/open approach patients	Confidence interval	*p* value
Posterior approach	3.43426	[0.8365153, 14.09913]	0.087
Number of levels fused	0.6079009	[0.4340611, 0.8513629]	0.004
Staged procedure	0.6507177	[0.1749019, 2.420978]	0.5186
Osteotomy	0.2604895	[0.03313165, 2.048035]	0.1703
Decompression	0.3597285	[0.04548798, 2.844809]	0.3127
Allograft	0.6491885	[0.2098068, 2.008732]	0.4505
Autograft	0.1382386	[0.01769585, 1.079909]	0.0289
Operation involving Thoracic spine	0.1539582	[0.008965574, 2.643795]	0.0805
